# Cervical Spine Mechanism for Reproduction of the Biomechanical Behaviours of the Human Neck during Rotation-Traction Manipulation

**DOI:** 10.1155/2017/5829048

**Published:** 2017-11-12

**Authors:** Yuancan Huang, Shuai Li, Minshan Feng, Liguo Zhu

**Affiliations:** ^1^School of Mechatronical Engineering, Beijing Institute of Technology, 5 South Zhongguncun Street, Haidian District, Beijing 100081, China; ^2^Wangjing Hospital, China Academy of Chinese Medicine Sciences, Beijing 100102, China

## Abstract

Rotation-traction (RT) manipulation is a commonly used physical therapy procedure in TCM (traditional Chinese medicine) for cervical spondylosis. This procedure temporarily separates the C3 and C4 cervical vertebrae from each other when a physician applies a jerky action while the neck is voluntarily turned by the patient to a specific position as instructed by the physician, where the cervical vertebrae are twisted and locked. However, a high rate of cervical injury occurs due to inexperienced physician interns who lack sufficient training. Therefore, we developed a cervical spine mechanism that imitates the dynamic behaviours of the human neck during RT manipulation. First, *in vivo* and *in vitro* experiments were performed to acquire the biomechanical feature curves of the human neck during RT manipulation. Second, a mass-spring-damper system with an electromagnetic clutch was designed to emulate the entire dynamic response of the human neck. In this system, a spring is designed as rectilinear and nonlinear to capture the viscoelasticity of soft tissues, and an electromagnetic clutch is used to simulate the sudden disengagement of the cervical vertebrae. Test results show that the mechanism can exhibit the desired behaviour when RT manipulation is applied in the same manner as on humans.

## 1. Introduction

Cervical spondylosis is a general and nonspecific medical term referring to degenerative changes that develop either spontaneously with age or secondarily as the result of trauma or other pathological conditions. More specifically, by the age of 65 years, 95% of patients are affected by degenerative disorders of the spine [1]. In China, the incidence of such disorders is between 3.8% and 17.6% of the total population [2]. Treatments for cervical spondylosis are typically conservative in nature, and physical modalities are the preferred treatments for spine-related disorders [3]. RT manipulation is an effective physical therapeutic modality for cervical spondylosis with mild symptoms. As commonly practiced in TCM hospitals throughout China, this procedure consists of a jerky action applied by a physician on the patient's neck. Well-controlled clinical studies conducted by Wangjing Hospital of the Chinese Academy of Traditional Chinese Medicine have shown that RT manipulation may loosen adhesions within the dural sleeves, reduce compression and irritation of discs, and improve circulation in the epidural space of the neck and is relatively more effective for cervical radiculopathy [4]. However, inexperienced physician interns who perform this therapy are prone to inadvertent errors, resulting in medical malpractice events ranging from soft tissue contusion to serious spine injury, even when under the instruction of skilled physicians. Therefore, a device that simulates the biomechanical behaviours of the human neck during RT manipulation and can objectively evaluate RT manipulation performance would be beneficial for training physicians and for spreading this traditional therapy around the world.

From an engineering point of view, living tissue is a load-transmitting mechanism [5]. Therefore, mechanical principles (e.g., statics, strength of materials, and stress analysis) can be applied to solve the biological problems of the cervical spine. In the literature, the biomechanical behaviours of the human neck have been studied using both *in vivo* and *in vitro* approaches [6, 7]. In the *in vivo* approach, desired mechanical parameters such as displacement, velocity, acceleration, and external forces applied on subjects by clinicians are directly measured using dedicated sensor systems. In contrast, *in vitro* approaches are model-based. Three types of methods are used to study the biomechanics of the human cervical spine: mathematical computation models, such as finite element analysis [8, 9]; anthropometric test dummies [10], such as Hybrid III; and whole cadavers [11, 12] or isolated whole cervical spine (WCS) specimens [13–16].

In response to the demand for RT manipulation training devices, we designed a cervical spine mechanism with three degrees of freedom (DOF: two revolute and one prismatic) to replicate the biomechanical behaviours of the cervical spine during RT manipulation and to automatically evaluate physician performance during execution of RT manipulation in the same manner as on a human. The three main contributions of this work are described as follows: the biomechanical features of the cervical spine are extracted from the *in vivo* and *in vitro* experimental data, the combination of a nonlinear spring and an electromagnetic clutch is designed to capture the biomechanical behaviours of the cervical spine during RT manipulation, and the cervical spine mechanism is developed to aid inexperienced practitioners in improving their skills via objective evaluation.

The remainder of this paper is organized as follows. The biomechanical parameters of the cervical spine are extracted from *in vivo* and *in vitro* experimental data in Section 2. An innovative mass-spring-damper model with an electromagnetic clutch is proposed to capture the biomechanical features of the cervical spine. A lumped parameter model of the cervical spine and a rectilinear nonlinear spring are presented in the subsequent section. Mechanism design and computer simulation are performed in Section 4. Finally, a cervical spine mechanism system is built that can emulate the abrupt acceleration change during the application of jerky manipulation. Moreover, experiments are implemented to verify the effectiveness of the cervical spine mechanism system for training physician interns.

## 2. Biomechanical Parameters of the Cervical Spine during RT Manipulation

### 2.1. RT Manipulation Operation

**Figure 1:**
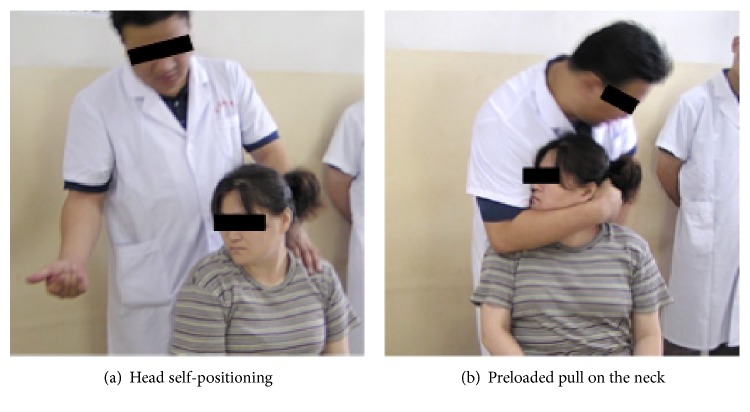
Scenario in which RT manipulation is executed by an experienced physician.

The RT manipulation includes four steps: head self-positioning, preloaded pull on the neck, jerky action, and restitution. First, the patient sits upright in a chair and relaxes the body. Under the physicians' instruction, the patient voluntarily turns his/her head to the left or right to its physiological limit, lowers the chin against the chest, and turns again in the same direction as in the first turn until the head cannot move further. Second, the physician pulls up gently and slowly on the patient's head with a forearm while tightly holding the mandible to find the position at which the cervical vertebrae are mutually twisted and locked and to determine the amount of force that should be exerted such that the cervical facets are instantly detached from their capsules without injury. Third, to prepare for the jerky action, the physician retracts slightly from the twisted and locked position and applies a high-speed and low-amplitude upward jerk. This lashing movement is executed together with an audible release or a cracking sound, which announces a successful manipulation. Finally, the physician loosens the forearm gradually such that the patient's neck can spontaneously return to its original state.  Figure 1 shows a scenario in which RT manipulation is executed by an experienced physician.

### 2.2. Measuring Biomechanical Parameters *In Vivo*

In RT manipulation, only vertically oriented force is expected, as other forces or torques might cause injuries. Therefore, the one-dimensional vertical force is measured to represent a standard RT manipulation.

**Figure 2:**
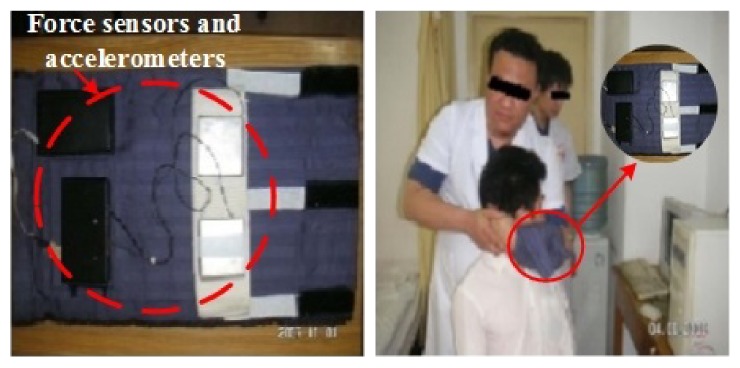
Dedicated measurement device for *in vivo* experiments and experimental snapshot.

During the *in vivo* experiments, a dedicated measurement device that includes force sensors and accelerometers was used to detect the vertical force exerted on the neck by the physician and the induced acceleration of the head. Strain gauges were used as force sensors, and their resistance variations were captured by the output of a Wheatstone bridge. The dual-axis acceleration sensor ADXL202 can measure both dynamic and static acceleration with digital signal output, and its maximum measurement range is ±2 ~ 10 g.  Figure 2 shows the dedicated measurement device and an experimental snapshot.

**Figure 3:**
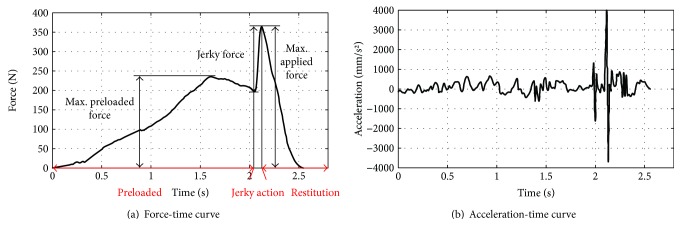
Force- and acceleration-time curves in a standard RT manipulation.

The force- and acceleration-versus-time curves in a standard RT manipulation are plotted in Figure 3, from which we conclude that the preloaded phase normally lasts 2 ~ 5 s, while, in contrast, the jerky action lasts only approximately 110 ms. Two peaks exist in the force-time waveform, and the second peak is much higher than the first. The traction force increases gradually at first, prior to the first peak, and subsequently decreases to a certain extent in the retraction for the jerky action. Near the second peak, the traction force varies steeply in a notably short time. The value of the second peak is defined as the maximum applied force. The head acceleration jumps positively to its maximum in response to the maximal applied force and then decreases rapidly when the exerted force disappears. Mathematically, the jerky action can be described as a high-speed, low-amplitude, one-dimensional impulse motion.

A number of tests on subjects were applied in a systematic manner by Wangjing Hospital, such that the evaluation criteria for standard RT manipulation have been deduced. After statistical analysis with respect to obese, overweight and normal-weight patient groups, the averages and variances of the biomechanical parameters are listed in Table 1.

**Figure 4:**
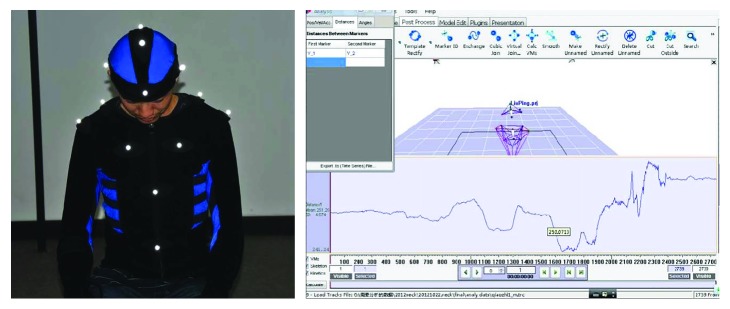
Measuring the displacement-time curve using OptiTrack S250e.

To analyze the variable stiffness characteristics of the cervical spine, the OptiTrack S250e three-dimensional (3D) motion-capture system was used to measure the displacement of the head during RT manipulation. The marker points (trackers) are arranged on the subject's head and trunk, as shown in Figure 4.

**Figure 5:**
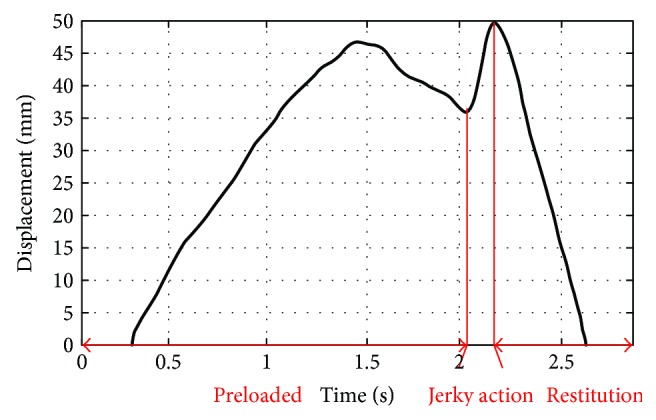
Displacement-time curve with time alignment.

After data processing and time alignment with the measurements from the dedicated measurement device, the displacement-time curve was plotted as shown in Figure 5, from which we can read the maximum displacement and the displacement related to the preloaded phase.

### 2.3. Measuring the Maximum Allowed Acceleration on WCS Specimens

**Figure 6:**
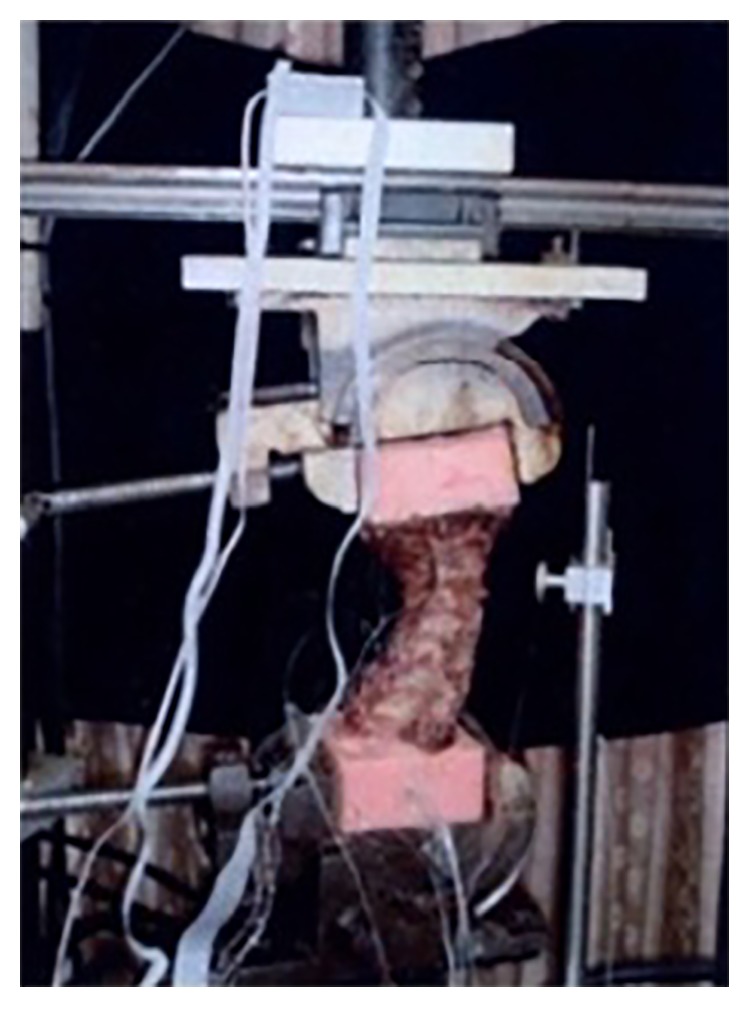
The maximum allowable acceleration of the cervical spine measured with the Zwick Roell BX1-EZ005.

Limiting the maximum allowable acceleration on the cervical spine is crucially important to prevent physical injuries during RT manipulation, and such acceleration can be measured *in vitro* by using an axial material testing device (Zwick Roell BX1-EZ005 A4K-000) to simulate the jerky action on a WCS cadaveric specimen (see Figure 6).

The WCS specimens were mounted in the twisted and locked position, and jerky forces with magnitudes of 50 N, 150 N, and 250 N were loaded over time intervals of 70 ms, 110 ms, and 150 ms, respectively. The acceleration are given in Table 2, which shows that the maximum allowable acceleration for safe RT manipulation is approximately 4600 mm/s^2^.

## 3. Cervical Spine Model

### 3.1. Lumped Parameter Description of the Cervical Spine

Despite their distributed nature, soft tissues such as skin, muscle, cartilage, and ligaments are typically modelled using lumped parameter models. In general, these materials can be treated as exhibiting linear behaviour if the strain remains small. If the strain does not exceed 1 mm, the mechanical behaviour is considered to be linearly viscoelastic [17] and is modelled reasonably well by parallel or/and serial combinations of linear springs and linear dashpots, such as the Kelvin-Voigt and Maxwell models and their variants [18, 19]. To describe the contact behaviour of soft tissues in situations where viscous effects are substantial, Hunt and Crossley [20] argued that a model will agree better with physical intuition if the damping coefficient is dependent on relative penetration. Further studies showed that the Hunt-Crossley model is consistent with the notion of the coefficient of restitution-characterised energy loss during impact [21].

**Figure 7:**
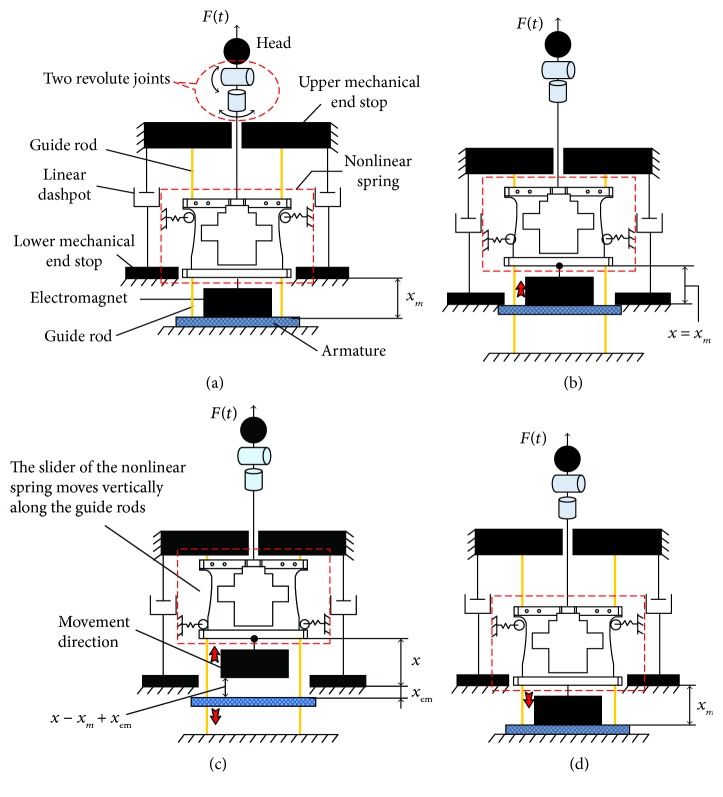
Cervical spine model and its reciprocating motion: (a) initial position; (b) maximal preloaded force is attained; (c) electromagnet is detached from the armature by the jerky action; (d) restoration to the initial position.

Unfortunately, existing models cannot be directly adopted to capture the behaviour of the cervical spine during RT manipulation; as the strain is far greater than 1 mm, the viscous effect is less insignificant due to the low speed in the preloaded phase, and a sliding phenomenon occurs in the aftermath of the jerky action. In terms of the displacement curve in Figure 5, the majority of the displacement results from the preloaded phase. Together with the low-speed feature, the behaviour of the cervical spine in the preloaded phase is reasonably captured by a nonlinear hard spring positioned in parallel with linear dashpots. Furthermore, an electromagnetic force limiter emulates the facet-sliding phenomenon during the jerky action. When the force exerted by a trainee exceeds the attractive force of the electromagnet, the electromagnet is detached from the armature, and thus a spiky acceleration emerges until the movement along the guide rods is stopped by the upper mechanical end stop. The cervical spine model and its reciprocating motion are illustrated schematically in Figure 7, where the head turns automatically to set the head at the “twisted and locked” position.

### 3.2. Dynamics Analysis

The dynamics of the cervical spine model is described as follows:

(i) In the preloaded phase,
  (1) $$\begin{array}{c} F\left(t\right)=\left(m_{1}+m_{2}\right)\left(\ddot{x}+g\right)+2\left(\mu_{1}+\mu_{2}\right)\dot{x}+F_{spring}\left(x\right),\;t\leq t_{jerk},F\left(t\right)<F_{m}. \end{array}$$
(ii) In the jerky action phase, three cases exist.

*Case 1.* The armature does not contact the lower mechanical end stop at all, or it contacts the lower mechanical end stop, but *F*(*t*) ≤ *F*_*m*_, and we obtain

(2) $$\begin{array}{c} F\left(t\right)=\left(m_{1}+m_{2}\right)\left(\ddot{x}+g\right)+2\left(\mu_{1}+\mu_{2}\right)\dot{x}+F_{spring}\left(x\right),\;t>t_{jerk},F\left(t\right)<F_{m},x\leq x_{m}. \end{array}$$

*Case 2. F*(*t*) > *F*_*m*_. The electromagnet is detached from the armature, and the attractive force decreases gradually. We obtain

(3) $$\begin{array}{c} F\left(t\right)=\left(m_{1}+m_{2}\right)\left(\ddot{x}+g\right)+2\mu_{1}\dot{x}+F_{spring}\left(x\right)+F_{em}\left(x-x_{m}+x_{em}\right),\;t>t_{jerk},F\left(t\right)>F_{m},x\leq x_{\text{max}}, \end{array}$$

and the dynamics of the armature are governed by

(4) $$\begin{array}{c} F_{em}\left(x-x_{m}+x_{em}\right)=m_{2}\left({\ddot{x}}_{em}+g\right)+2\mu_{2}{\dot{x}}_{em}. \end{array}$$

*Case 3.* The moving component is stopped by the upper mechanical end stop; in which case, we obtain

(5) $$\begin{array}{c} F\left(t\right)=m_{1}g+F_{spring}\left(x\right)+F_{em}\left(x-x_{m}+x_{em}\right),\;t>t_{jerk},x=x_{\text{max}}. \end{array}$$

(iii) In the restitution phase, two cases exist.

*Case 1.* The electromagnet is detached from the armature when it moves downwards, and we obtain

(6) $$\begin{array}{c} F\left(t\right)=m_{1}\left(g-\ddot{x}\right)-2\mu_{1}\dot{x}+F_{spring}\left(x\right)+F_{em}\left(x-x_{m}+x_{em}\right). \end{array}$$

*Case 2.* The electromagnet is attached to the armature, and they move downwards together until the armature returns to its initial position, and we obtain

(7) $$\begin{array}{c} F\left(t\right)=\left(m_{1}+m_{2}\right)\left(g-\ddot{x}\right)-2\left(\mu_{1}+\mu_{2}\right)\dot{x}+F_{spring}\left(x\right). \end{array}$$

### 3.3. Electromagnet

**Figure 8:**
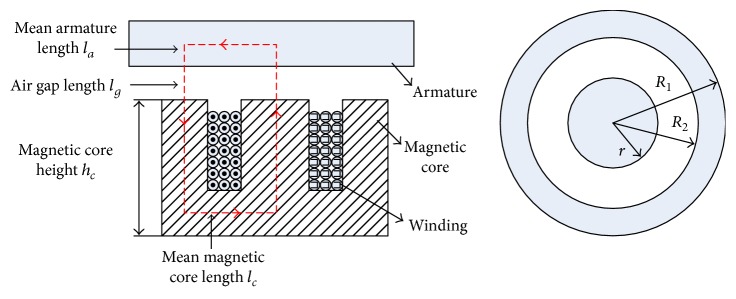
Schematic diagram of the electromagnet.

Consider the cylindrical electromagnet shown in Figure 8. We assume that the magnetic flux density is uniform in the electromagnet, air gap, and armature and that the relationships between the magnetic field intensity and the magnetic flux density are linear. Neglecting the leakage of magnetic flux, using Gauss' Law, we obtain

(8) Bc=BgAgAc,Ba=BgAgAa=B,g

where *B*_*c*_, *B*_*g*_, and *B*_*a*_ are the magnetic flux densities in the electromagnet, the air gap, and the armature, respectively, and *A*_*c*_ = *π*(*r*^2^ + *R*_1_^2^ − *R*_1_^2^) and *A*_*g*_ = *A*_*a*_ = *πR*_1_^2^ are the corresponding cross-sectional areas.

Similarly, by *Ampere's Law*, we obtain

(9) $$\begin{array}{c} \begin{array}{l} NI=H_{c}l_{c}+H_{g}l_{g}+H_{a}l_{a}\\ =\frac{B_{c}l_{c}}{\mu_{c}}+\frac{2B_{g}l_{g}}{\mu_{g}}+\frac{B_{a}l_{a}}{\mu_{a}}\\ =\left(\frac{A_{g}l_{c}}{A_{c}\mu_{c}}+\frac{2l_{g}}{\mu_{0}}+\frac{l_{a}}{\mu_{a}}\right)B_{g}, \end{array} \end{array}$$

where *N* is the number of turns in the winding, *I* is the current in the wire, *μ*_*c*_ and *μ*_*a*_ are the relative magnetic permeability of the electromagnetic core and the armature, respectively, and *μ*_0_ = 4*π* × 10^−7^ N/A^2^ is the permeability of free space. In addition, *l*_*c*_ = 2*h*_*c*_ + *R*_1_ and *l*_*a*_ ≈ *R*_1_ are the mean lengths of the magnetic core and the armature, respectively, and *l*_*g*_ is the air gap length. Therefore, the magnetic flux density in the air gap *B*_*g*_ is

(10) $$\begin{array}{c} B_{g}=\frac{NI}{\left(A_{g}l_{c}/A_{c}\mu_{c}\right)+\left(2l_{g}/\mu_{0}\right)+\left(l_{a}/\mu_{a}\right)}, \end{array}$$

and the electromagnetic force in the air gap is computed by

(11) $$\begin{array}{c} F_{em}=\frac{B_{g}^{2}A_{g}}{2\mu_{0}}. \end{array}$$

### 3.4. Rectilinear Nonlinear Spring

**Figure 9:**
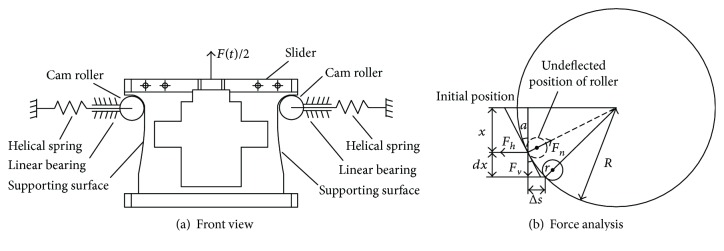
Front view and force analysis of the nonlinear spring.

The nonlinear spring is composed of a slider with axially symmetric curvilinear supporting surfaces and four spring-bearing roller sets arranged in radial symmetry, as shown in Figure 9(a). The slider is able to freely move up and down along the guide rods, and the cam rollers are pressed tightly against the curvilinear surfaces by helical springs via linear bearings. Several curvilinear surfaces with different curvatures may be tangentially joined together to form the supporting surfaces.

We take as an example a component of the supporting surface with curvature radius *R*. The force analysis on a cam roller is given in Figure 9(b), where *F*_*h*_ represents the restoring force from the helical springs and *F*_*n*_ and *F*_*v*_ are the normal and tangential components of the supporting force, respectively. Let *r* denote the radius of the cam roller, *x* the displacement of the slider relative to the reference position, *m* the mass of the slider and the head, and *F*(*t*) the external force. The deflected angle is thus calculated by

(12) $$\begin{array}{c} \alpha =\arcsin\left(\frac{x}{R}\right). \end{array}$$

First, we obtain the normal and tangential force balance relations

(13) $$\begin{array}{c} F_{n}\sin\alpha =F_{v},\\ F_{n}\cos\alpha =F_{h}, \end{array}$$

from which it follows that *F*_*v*_ = *F*_*h*_tan*α*. However, because the displacement of the helical spring is *s* = *R*(1 − cos*α*)*x*, we write

(14) $$\begin{array}{c} F_{h}=k\left(\sigma +s\right)=k\left[\sigma +R\left(1-\text{cos}\alpha \right)\right], \end{array}$$

where *σ* and *k* are the preloaded deformation and stiffness of the helical spring, respectively. Thus, the resilient force, excluding the gravity bias, satisfies the equation

(15) $$\begin{array}{c} F_{r}=4F_{h}\text{tan}\alpha =4k\left[\sigma +R\left(1-\text{cos}\alpha \right)\right]\text{tan}\alpha , \end{array}$$

and hence, its stiffness is

(16) $$\begin{array}{c} K=\frac{{dF}_{r}}{dx}=4k\left[-1+\frac{R+\sigma }{R{\text{cos}}^{2}\left(\alpha \right)}\right]=4k\left[-1+\frac{R+\sigma /R}{{\left(1-x^{2}/R^{2}\right)}^{3/2}}\right]. \end{array}$$

From the above equation, we know that the stiffness feature can be changed to a certain extent with variation of the preloaded deformation. This property is used to generate the different stiffness-displacement curves on identical supporting surfaces corresponding to the obese, overweight, and normal weight patient groups.

## 4. Design and Computer Simulation

### 4.1. Rectilinear Nonlinear Spring

The stiffness feature of the nonlinear spring dominates the behaviours of the cervical spine mechanism in the preloaded phase, where the inertia force and the viscous effect can be neglected. From Figure 5 and Table 1, the maximum displacement of the neck in RT manipulation is approximately 50 mm, and the maximum force applied by physicians is less than 450 N in a worst-case scenario, which is commensurate with the physiological pull tolerance of the human cervical spine [22]. The maximum resilient force of the nonlinear spring is set to the maximum preloaded force, that is, *F*_spring_ = 280 N at *x* = 40 mm. To adapt to parameters of the obese, overweight, and normal-weight patient groups, the preloaded deformation of the helical spring *σ* is adjusted to generate the different stiffness properties of the nonlinear spring, with a given *σ*_max_ = 6 mm.

**Figure 10:**
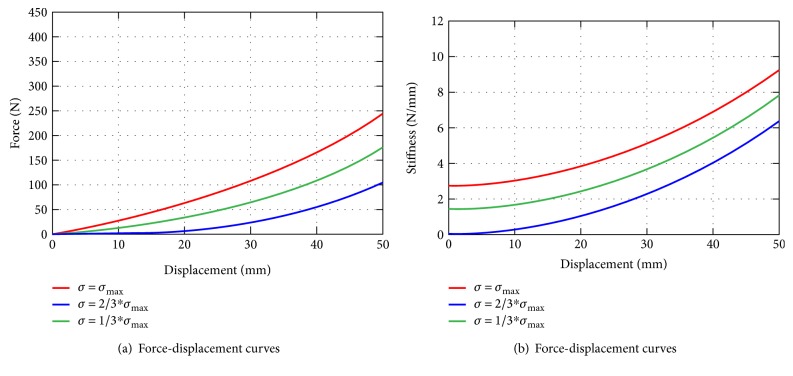
Force- and stiffness-displacement curves with respect to *σ* = 1/3*σ*_max_, 2/3*σ*_max_, and *σ*_max_.

Three different values of *σ* = 1/3*σ*_max_, 2/3*σ*_max_, and *σ*_max_ were selected with respect to the obese, overweight, and normal-weight cases, respectively, and the corresponding force- and stiffness-displacement curves are plotted in Figure 10.

According to the stiffness formula of rectilinear nonlinear spring by theoretical deduction, the size of the cam roller is not related to the stiffness, whose effect is just force transmission and passive accompany movement; therefore, the given cam roller radius is *r* = 10 mm. As for the determination of the radius *R*, according to the maximum value of the three parameters (*F*_r,max_, *σ*_max_, and *x*_max_) determined in the abovementioned statement, the expression of the rectilinear nonlinear spring stiffness can be reduced to the one only related to *R* and *k*; in general, the spring used to transfer force needs to have large enough stiffness. Through repeated selection and checking, when *k* = 35.5 ~ 40 N/mm, it can satisfy the strength condition and the working travel condition, and finally, *k* = 35.5 N/mm is selected preferably, and the value of *R* can be obtained, *R* = 280 mm.

### 4.2. Electromagnet Selection

**Figure 11:**
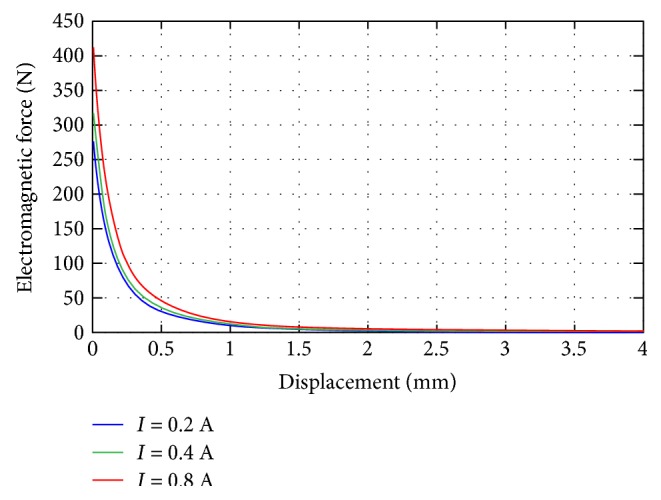
Force-displacement curves of the electromagnet with respect to the obese, overweight, and normal-weight groups.

The electromagnet imitates the sliding phenomenon during the jerky action, and its maximum attractive force must thus be greater than the maximum preloaded force but less than the maximum applied force corresponding to the obese group, which was selected as 420 N. The electromagnet core is made of pure iron with *μ_c_* = 4000, and the armature is constructed from carbon steel with *μ_a_* = 100. The other parameters of the electromagnet were chosen as follows: *N* = 2, 000turns, *h_c_* = 27 mm, *R_1_* = 25 mm, *R_2_* = 23 mm, *r* = 20 mm, and *l_g_* = 0 ~ 4 mm. The attractive force of the electromagnet is varied by adjusting the current in the windings. The force-displacement relationships of the electromagnet with respect to the obese, overweight, and normal-weight patient groups are plotted in Figure 11, and they initially begin at 420 N, 325 N, and 283 N, respectively.

### 4.3. Computer Simulation

Other parameters were determined by trial and error to sculpt the force-displacement curves in terms of those shown in Figures 3 and 5. Specifically, we used, *m_1_* = 9 kg (including the standard head weight of 7 kg for adults and the slider weight of 2 kg), *m_2_* = 0.42 kg, *R* = 280 mm, *k* = 35.5 N/mm, and a mechanically limited maximum stroke of the slider of 53 mm, making *x*_max_ = 55 mm if the damping coefficients of the two commercial linear dashpots and guide rods are 150 N·s/mm and 2 N·s/mm, respectively. Taking the case of the obese group as an example, the dynamic behaviours of the cervical spine mechanism were simulated according to the dynamics described in the preceding sections. The applied force for the obese group during a standard RT manipulation *F*(*t*) is obliged to comply with the following rules, which were extracted from a number of experimental data in the biomechanics study:

- The maximum preloaded, jerky, and maximum applied forces are, respectively, confined to 230 ± 50 N, 174 ± 50 N, and 362 ± 75 N.
- The slope of the force-time curve in the preloaded phase *k*_pre_ should be limited to a range of 82 ~ 201 N/s, such that no abrupt acceleration occurs, and hence, patients do not feel discomfort during this phase.
- The burst duration of the jerky action must be less than 150 ms.
- At the end of the jerky action, the physician must move his/her arm downwards slowly and tightly against the patient's chin, such that patient's cervical spine gradually returns to its original position.

**Figure 12:**
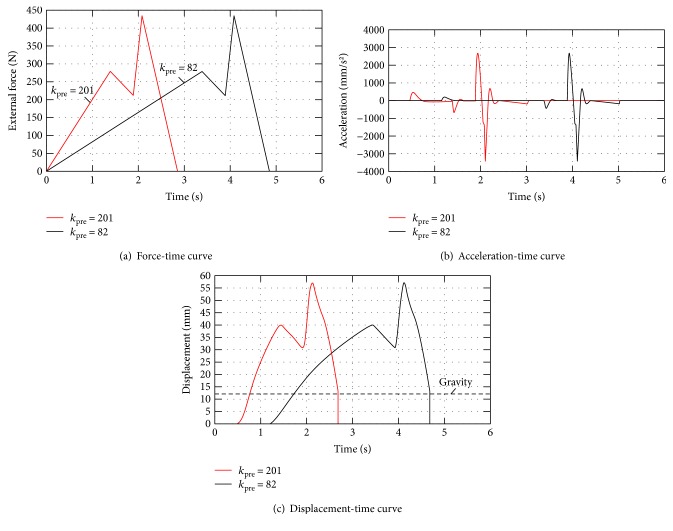
Force-, acceleration-, and displacement-versus-time curves during simulated RT manipulation on the cervical spine mechanism.

As shown in Figure 12, we can obtain the expected displacement- and acceleration-time curves using the cervical spine mechanism with the given parameters, and the applied force *F*(*t*) is constructed to conform to the listed rules.

## 5. Mechanical Implementation

### 5.1. Rectilinear Nonlinear Spring

**Figure 13:**
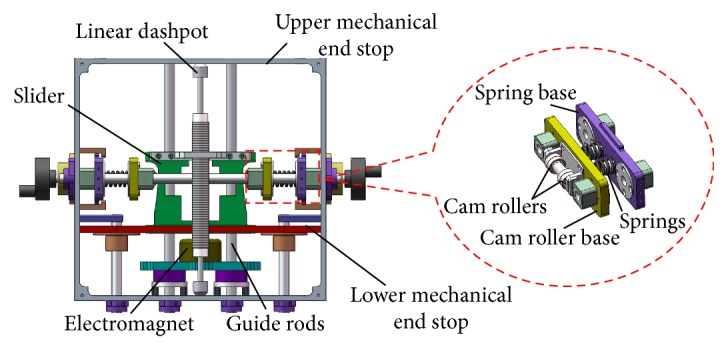
Nonlinear spring mechanism.

Figure 13 shows a schematic diagram of the nonlinear spring mechanism. The slider can only slide vertically along the guide rods, and the cam rollers are pressed tightly against the supporting surfaces by the helical springs, whose preloaded deformation *σ* is adjusted via a hand wheel using the screw-thread fit of nuts and bolts to generate the different stiffness properties of the nonlinear spring so as to imitate the obese, overweight, and normal-weight patient groups; the heavier the weight group is set by the mechanism, the greater the force the operators need to exert.

### 5.2. Cervical Spine Mechanism

**Figure 14:**
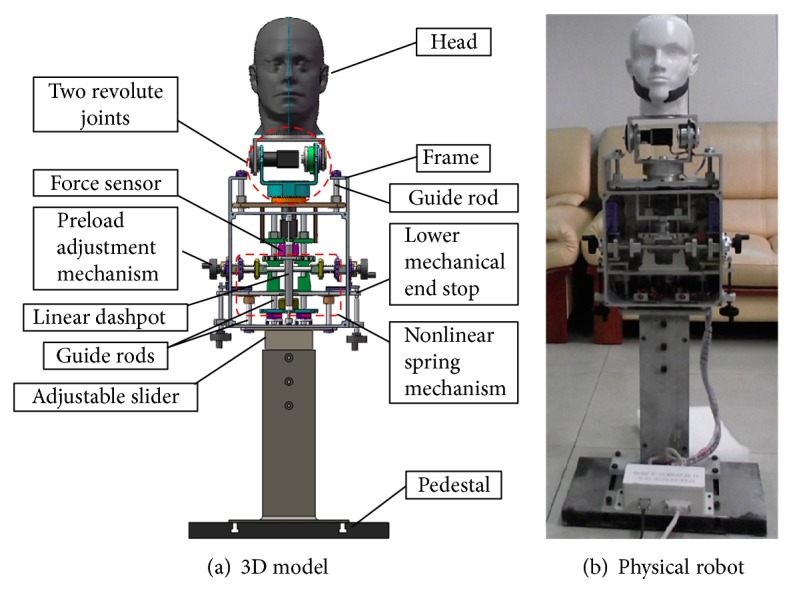
Cervical spine mechanism.

As shown in Figure 14, the 3-DOF cervical spine mechanism consists of a head, a torso, and a pedestal. To imitate head movements, the 2-DOF head automatically turns 70° leftwards or rightwards, bends 30° downwards, and again turns a further 10° in the same direction as the initial rotation. The torso contains a frame, two linear dampers, an electromagnetic clutch, and a rectilinear nonlinear spring. A force sensor is placed between the head mechanism and the nonlinear spring, which measures the force exerted on the head by trainees. The adjustable slider is used to adjust the height of the mechanism, so as to be suitable for operators of different height.

### 5.3. Impact Load Strength Analysis

**Figure 15:**
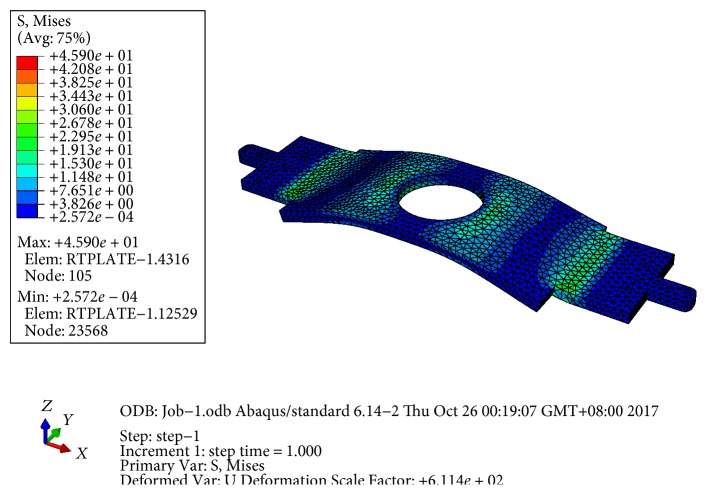
Stress distribution diagram as plotted by Abaqus.

In the jerky action phase, a high-impact load occurs due to abrupt collision with the lower mechanical end stop, a thin, rectangular carbon steel plate. The electromagnet automatically disengages from the armature if the contact force is greater than or equal to the electromagnetic attractive force. Therefore, the maximum impact force is reasonably assumed to be the maximum attractive force *F*_em_ = 400 N distributed uniformly over an annular contact region. Static failure analysis is done by the commercial finite element analysis software Abaqus, the stress distribution diagram of the thin plate is plotted in Figure 15, where the Young's modulus of carbon steel is 210GPa, Poisson's ratio is 0.3, the ultimate tensile strength is *S*_ut_ = 600 MPa, and the yield strength is *S_y_* = 355 MPa. A tetrahedral mesh was applied, and the boundary conditions and loading conditions were set as follows. The thin plate is fixed at both ends, and impact loading is uniformly distributed over the circular annulus in the vicinity of the circular hole.

Based on the Mises-Hencky theory, also known as the maximum distortion energy theory, when the effective stress is equal to or greater than the yield strength of the material, the material is invalid. In terms of the principal stresses *σ*_1_, *σ*_2_, and *σ*_3_, the effective stress *σ*_vonMises_ is expressed as

(17) $$\begin{array}{c} \sigma_{vonMises}=\frac{1}{\sqrt{2}}\sqrt{{\left(\sigma_{1}-\sigma_{2}\right)}^{2}+{\left(\sigma_{2}-\sigma_{3}\right)}^{2}+{\left(\sigma_{1}-\sigma_{3}\right)}^{2}}. \end{array}$$

From Figure 15, the maximum effective stress *σ*_vonMises_ is 45.9 MPa. Thus, the safety factor SF is

(18) $$\begin{array}{c} SF=\frac{S_{y}}{\sigma_{vonMises}}=\frac{355\;Mpa}{45.9\;Mpa}=7.7>1, \end{array}$$

which indicates that the thin plate sufficiently meets the strength requirement of the Mises-Hencky theory.

#### 5.3.1. Fatigue Failure

Although the static strength requirement was met, we had to consider the need for regular use, that is, the fatigue life of the thin plate. The fatigue limit strength *S*_*e*_ satisfies the following formula:

(19) $$\begin{array}{c} S_{e}=c_{surf}c_{size}c_{load}c_{misc}S_{e^{\prime }}, \end{array}$$

where *S*_*e*_ represents the modified endurance limit, *S*_*e*′_ is the theoretical endurance limit and *c*_surf_, *c*_size_, *c*_load_, and *c*_misc_ are correction factors. Often, the value of *S*_*e*′_is unavailable, and a rough approximation must be generated to obtain an estimate for the design calculations. The following relationships [23] were chosen for the initial approximation. For carbon steel

(20) $$\begin{array}{c} S_{e^{\prime }}=\left\{\begin{array}{ll} 0.5S_{ut}, & for\;S_{ut}<200\;kpsi\;\left(1400\;Mpa\right),\\ 0.5\;kpsi, & for\;S_{ut}\geq 200\;kpsi\;\left(1400\;Mpa\right). \end{array}\right. \end{array}$$

**Figure 16:**
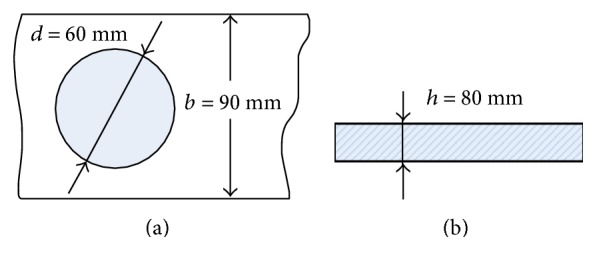
Dimensions of the thin plate.

Hence, we obtain *S*_*e*′_ = 0.5*S*_ut_ = 0.5 × 600 MPa = 300 MPa. The correction factors are described below:

(i) Surface factor: the surface finish is one of the most critical aspects to consider in fatigue life prediction. Shigley [24] suggests the expression:
  (21) $$\begin{array}{c} c_{surf}=\left\{\begin{array}{ll} {aS}_{ut}^{b} & if\;{aS}_{ut}^{b}<1\\ 1 & if\;{aS}_{ut}^{b}\geq 1 \end{array}\right.. \end{array}$$
  Because the surface of the thin plate is machined, *a* = 4.45 MPa and *b* = −0.265 were selected, with reference to the study of Howell [23]. We thus obtain *c*_surf_ = 0.8.
(ii) Size factor: the component under analysis typically has a different size than the standard fatigue specimen. If the cross-sectional area is larger, a larger probability of surface imperfection exists. Shigley recommend the following approximation [24]:
  (22) $$\begin{array}{c} c_{size}=\left\{\begin{array}{ll} 1, & for\;d<2.79\;mm,\\ {\left(\frac{d}{7.62}\right)}^{-0.1133}, & for\;d\;is\;in\;millimeters\;and\;2.79\leq d\leq 51\;mm,\\ 0.6, & for\;d>51\;mm. \end{array}\right. \end{array}$$
  Because this equation considers the size effect, assuming a circular cross-section in rotation, bending, or torsion, an equivalent diameter *d*_*e*_ for a nonrotating rectangular cross-section with side dimensions *b* and *h* is
  (23) $$\begin{array}{c} d_{e}=0.808\sqrt{bh}. \end{array}$$
  For the thin plate, we used *c*_size_ = 0.86.
(iii) Load factor: because only bending exists in the thin plate, it follows that *c*_load_ = 1 [25].
(iv) Stress concentration effects: most fatigue failure occurs at a stress concentration, and its effect is expressed mathematically by
  (24) $$\begin{array}{c} c_{misc}=\frac{1}{K_{f}}, \end{array}$$
  where *K*_*f*_ is the stress concentration coefficient. In reference to the mechanical design manual [26], the stress concentration factor of a finite-width plate with a central hole is
  (25) $$\begin{array}{c} K_{f}=\left[1.793+\frac{0.131}{d/h}+\frac{2.052}{{\left(d/h\right)}^{2}}-\frac{1.019}{{\left(d/h\right)}^{3}}\right]\times \left[1-1.04\left(\frac{d}{b}\right)+1.22{\left(\frac{d}{b}\right)}^{2}\right], \end{array}$$
  where the diameter of the central hole is *d* = 60 mm, the width of the thin plate is *b* = 95 mm, and the height is *h* = 8 mm, as shown in Figure 16. Thus, *K_f_* = 1.57 and *c*_misc_ = 1/*K*_*f*_ = 0.64.

Finally, we obtain

(26) $$\begin{array}{c} \begin{array}{l} S_{e}=c_{surf}c_{size}c_{load}c_{misc}S_{e^{\prime }}\\ =0.8\times 0.86\times 1.0\times 0.64\times 300\;Mpa=132\;Mpa. \end{array} \end{array}$$

Because the maximum effective stress *σ*_vonMises_ = 45.9 MPa < *S*_*e*_ = 132 MPa, we conclude from the stress life curve [23] that the fatigue life cycles of the thin plate are greater than 10^6^. Moreover, because the impact load is a fluctuating load, the mean stress *σ*_*m*_ and the alternating stress *σ*_*a*_ of the thin plate can be computed as

(27) $$\begin{array}{c} \sigma_{m}=\sigma_{a}=\frac{\sigma_{vonMises}}{2}=\frac{45.9\;Mpa}{2}=22.95\;Mpa. \end{array}$$

According to the fatigue failure theory for incompletely reversed fluctuating loads [23],

(28) $$\begin{array}{c} SF=\frac{1}{\left(\sigma_{a}/S_{e}\right)+\left(\sigma_{m}/S_{ut}\right)}=4.7>1, \end{array}$$

which satisfies the requirement of fatigue strength.

## 6. Experimental Verification and Evaluation

**Figure 17:**
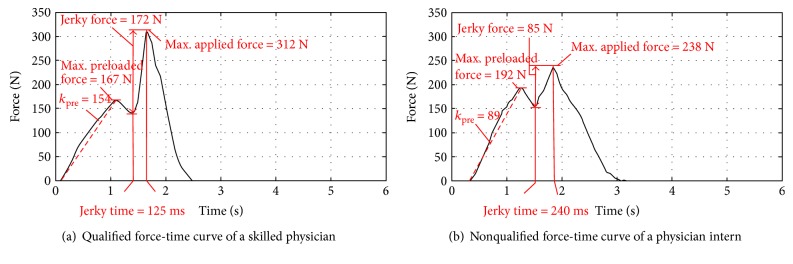
Qualified/nonqualified force-time curves.

Experiments were implemented to verify whether the cervical spine robot could reproduce the biomechanical properties of the human cervical spine and evaluate performance during RT manipulation training. First, 10 skilled physicians were invited to perform RT manipulation on the cervical spine robot. They all gave positive comments on manipulating similarity on the cervical spine robot and the human subject. For the RT manipulations accredited by the physicians, the acquired force-time curves show that the evaluated parameters, maximal preloaded force, jerky force, maximal force, and jerky action time, fall into the allowable ranges.  Figure 17(a) plots the force-time curve in one RT manipulation that the obese setup is selected, whose parameters are extracted from the force-time curve and are compared with the standard ranges in Table 3.

Figure 17(b) shows that an intern manipulation is not qualified because of the following shortcomings: (1) the jerky time is too long to generate the jerky action and (2) the jerky force is not strong enough to reach the upper mechanical end stop.

**Figure 18:**
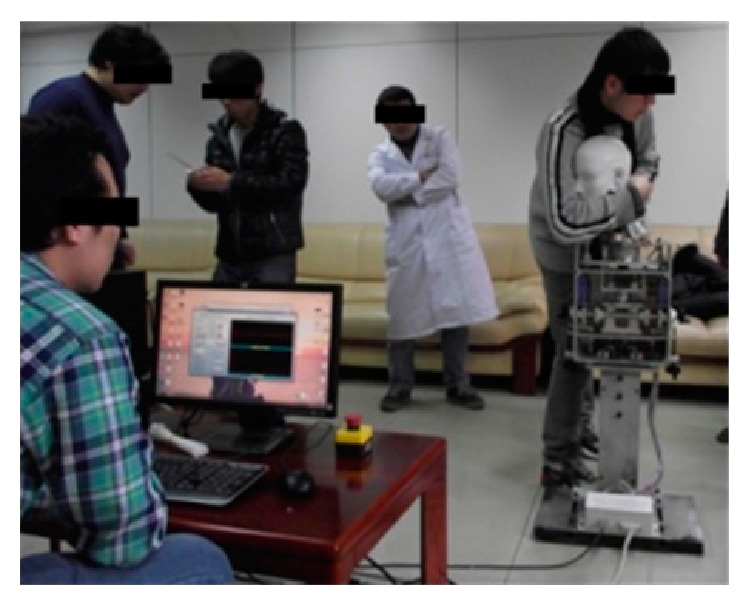
Intern physicians in evaluation of RT manipulation training.

To verify whether the mechanism is helpful for improving the manipulation capacity of physician interns, 30 interns were asked to execute RT manipulation on the cervical spine mechanism, as shown in Figure 18. Before training, the pass rates related to the four key parameters were less than 40%. After 100 repetitions of training, the pass rates all improved greatly, and one of the pass rates was greater than 80% (see additional details in Table 4). All of the subjects were orally interviewed after testing with respect to the similarity of manipulation between humans and the cervical spine mechanism, and 100% of subjects gave positive comments.

## 7. Conclusions

In this paper, the biomechanical properties of RT manipulation were determined from *in vivo* and *in vitro* measurements, and thus, a novel lumped parameter model that differs from the frequently-used Kelvin-Voigt and Hunt-Crossley models was proposed to capture the biomechanical properties of RT manipulation. In this cervical spine model, two innovations are worthy of emphasis. The first innovation is the use of a rectilinear nonlinear spring to capture the stiffness variation of the cervical spine due to large strain, and the second is the use of an electromagnet to imitate the sliding phenomenon that occurs during the jerky action of RT manipulation. A dynamic model of the cervical spine mechanism was presented and then verified by computer simulation. Finally, the cervical spinal mechanism system was implemented. The experimental results show that the cervical spinal mechanism can faithfully replicate the biomechanical properties of the human cervical spine during RT manipulation and is helpful in training of physician interns.

## Figures and Tables

**Table 1:** Averages and variances of biomechanical parameters.

	Obese	Overweight	Normal weight
Max. preloaded force (N)	230.3 ± 48.8	185.2 ± 41.8	153.1 ± 46.2
Jerky force (N)	173.5 ± 51.5	123.2 ± 33.9	117.2 ± 38.6
Max. applied force (N)	362.1 ± 74.4	285.6 ± 56.0	253.8 ± 54.0
Jerky duration (ms)	110 ± 20	110 ± 20	110 ± 20
Acceleration (mm/s^2^)	3836.3 ± 1262		
Velocity (mm/s)	203.0 ± 50.0		

**Table 2:** Acceleration readings from the *in vitro* experiments.

Load Force (N)	50	150	250
Time (ms)	70~150	70,110,150	70,110,150
Acceleration (mm/s^2^)	<784	1764 ± 882	3136 ± 1470
		1274 ± 372	2058 ± 686
		1078 ± 392	1470 ± 588

**Table 3:** Comparison of parameter values for correct manipulation and evaluation criteria.

	Preloaded force (N)	Jerky force (N)	Max force (N)	Jerky action time (ms)
Physician	167	172	312	125
Standard (obese)	230.3 ± 48.8	173.5 ± 51.5	362.1 ± 74.4	110 ± 20

**Table:** (a) Before training

	Preloaded force	Jerky force	Max force	Jerky time
Qualified	11	10	10	9
Unqualified	19	20	20	21
Pass rate (%)	36.67	33.33	33.33	30.00

**Table:** (b) After training

	Preloaded force	Jerky force	Max force	Jerky time
Qualified	24	25	25	27
Unqualified	6	5	5	3
Pass rate (%)	80.00	83.33	83.33	90.00

## Nomenclature

*m*_1_, *m*_2_: Masses of the slider and electromagnet, respectively
*F*_spring_: Resilient force of the nonlinear spring
*F*_em_: Electromagnetic attractive force exerted on the armature by the electromagnet, with *F_m_* being the threshold attractive force of the electromagnetic clutch
*x*: Displacement of the nonlinear spring slider with respect to the reference position, where *x_m_* and *x*_max_, respectively, represent the displacements that the external force attains based on the threshold force of the electromagnetic clutch and when the slider is stopped by the upper mechanical end stop
*x*_em_: Displacement of the armature with respect to the reference position when it detaches from the electromagnet
*μ*_1_, *μ*_2_: Damping coefficients of the linear dashpot and guide rod, respectively
*t*_jerk_: Starting time of the jerky action.
